# Genome Wide Identification of *Respiratory Burst Oxidase Homolog* (*Rboh*) Genes in *Citrus sinensis* and Functional Analysis of *CsRbohD* in Cold Tolerance

**DOI:** 10.3390/ijms23020648

**Published:** 2022-01-07

**Authors:** Yueliang Zhang, Yiwu Zhang, Li Luo, Chunyi Lu, Weiwen Kong, Libao Cheng, Xiaoyong Xu, Jihong Liu

**Affiliations:** 1 School of Horticulture and Plant Protection, Yangzhou University, Yangzhou 225009, China; 202000000071@just.edu.cn (Y.Z.); zyw_18796609849@163.com (Y.Z.); abcd17869359501@163.com (L.L.); luaulu@163.com (C.L.); wwkong@yzu.edu.cn (W.K.); lbcheng@yzu.edu.cn (L.C.); 2 Joint International Research Laboratory of Agriculture and Agri-Product Safety (The Ministry of Education), Yangzhou University, Yangzhou 225009, China; 3 Key Laboratory of Horticultural Plant Biology (MOE), College of Horticulture and Forestry Science, Huazhong Agricultural University, Wuhan 430070, China

**Keywords:** *Citrus*, respiratory burst oxidase homologs, cold stress, ROS

## Abstract

Respiratory burst oxidase homologs (Rbohs) are critical enzymes involved in the generation of reactive oxygen species (ROS) that play an important role in plant growth and development as well as various biotic and abiotic stresses in plants. Thus far, there have been few reports on the characterization of the *Rboh* gene family in *Citrus*. In this study, seven *Rboh* genes (*CsRbohA*~*CsRbohG*) were identified in the *Citrus sinensis* genome. The CsRboh proteins were predicted to localize to the cell membrane. Most CsRbohs contained four conserved domains, an EF-hand domain, and a transmembrane region. Phylogenetic analysis demonstrated that the CsRbohs were divided into five groups, suggesting potential distinct functions and evolution. The expression profiles revealed that these seven *CsRboh* genes displayed tissue-specific expression patterns, and five *CsRboh* genes were responsive to cold stress. Fourteen putative *cis*-acting elements related to stress response, hormone response, and development regulation were present within the promoters of *CsRboh* genes. The in-silico microRNA target transcript analyses indicated that *CsRbohE* might be targeted by csi-miR164. Further functional and physiological analyses showed that the knockdown of *CsRbohD* in trifoliate orange impaired resistance to cold stress. As a whole, our results provide valuable information for further functional studies of the *CsRboh* genes in response to cold stress.

## 1. Introduction

Plant NADPH oxidases (NOXs), known as Rbohs and encoded by *Rboh* genes, are homologs of the mammalian phagocyte NOXs catalytic subunit gp91^phox^ [1]. Rbohs often consist of six transmembrane central regions, two heme groups, cytosolic FAD- and NADPH-binding domains, and two EF-hand calcium-binding domains [2]. Through these domains, Rbohs can transfer electrons to extracellular O_2_ to produce O_2_^−^, and then rapidly converted into H_2_O_2_ by superoxide dismutase (SOD, EC 1.15.1.1). Increasing evidence suggests that Rboh-mediated ROS production is critical for plant growth and development as well as plant response to abiotic and biotic stresses [1,3,4]. Consequently, Rbohs are considered the important nodes in the ROS signaling network.

The first plant *Rboh* (*OsRbohA*) was identified in *Oryza sativa* [5], and more *Rboh* genes have been subsequently characterized in many other plant species. Rbohs are encoded by multiple genes, and the gene family has been identified and characterized in some plant species. So far, ten *Rboh* genes were found in *Arabidopsis* [6], nine in rice [7], seven in grapes [8], fourteen in *Nicotiana tabacum* [9], twenty-six in upland cotton [10], nine in apples [11], seven in strawberries [12], seven in jatropha [13], eight in cassava [14], fourteen in *Brassica rapa* [15], and eight in pepper [16]. Analysis of gene expression patterns may help to understand the possible function of Rboh family genes. In *Arabidopsis*, *AtRbohA-G* and *AtRbohI* were expressed in the roots, *AtRbohD* and *AtRbohF* in all tissues, and *AtRbohH* and *AtRbohJ* in pollen and stamen [6]. In rice, all *Rboh* genes, except for *OsRbohD* and *OsRbohH*, are constitutively expressed in roots, leaves, shoots, and calli [17]. In upland cotton, *Ghrboh15* and *Ghrboh16* were expressed constitutively in the stamen [10]. In pepper, *CaRbohA*, *CaRbohB*, and *CaRbohD* genes were highly expressed in the five tissues, but *CaRbohC*, *CaRbohF*, and *CaRbohG* genes were weakly expressed [16]. This differential tissue expression of *Rboh* genes has also been widely described in other plants [9,11,12,18], implying that the *Rboh* genes played important and diverse functional roles in different tissues.

Interestingly, *Rboh* genes are notably expressed under various abiotic stresses, especially low temperatures. For example, the transcription levels of *FvRbohA* and *FvRbohD* in strawberries were significantly induced by cold stress, followed by an enhancement in NADPH oxidase activity [12]. Five *GmRboh* genes (*A2*, *C1*, *F1*, *G*, and *H2*) and four *GmRboh* genes (*B1*, *B2*, *D2*, and *E2*) were up-regulated under cold stress in soybean roots and leaves, respectively [18]. The expression of seven *NtabRboh* genes (*A*, *B*, *C*, *M*, *N*, *O*, and *P*) were increased markedly following cold treatment in *Nicotiana tabacum* leaves [9]. In pepper, cold treatment significantly enhanced the expression of *CaRbohA* and *CaRbohB* genes [16]. These results indicate that *Rboh* genes are involved in cold stress responses, and the roles need to be further confirmed by transgenic studies in the future.

*Citrus* is one of the most important fruit crops planted worldwide, ranking first in production and yield among all fruit crops in 2019 (FAOSTAT data, https://www.fao.org/faostat/en/ accessed on 3 September 2021). As one of the sessile organisms, *Citrus* is frequently subjected to various biotic or abiotic stresses, including cold stress [19]. Considering that Rbohs play key roles in response to cold stress, the identification of *Citrus* Rbohs’ function offers an alternative way to enhance resistance to low-temperature stress. To the best of our knowledge, few efforts have been made to identify and characterize the *Rboh* gene family in *Citrus* [20]. In the current study, we identified the *Rboh* gene family members from the *Citrus sinensis* genome and systematically analyzed chromosomal localization, gene structures, conserved motifs, phylogenetic relationships, *cis-*acting elements, and potential microRNA (miRNA) target sites. Secondly, the expression profiles of *CsRboh* genes in different tissues and their response to cold stress were investigated by RNA-Seq data and quantitative real-time PCR (qRT-PCR) analysis, respectively. Finally, we investigated the function of CsRbohD associated with cold stress using virus-induced gene silencing (VIGS) system. As a whole, our results provide valuable information for further functional analysis of *CsRboh* genes in response to cold stress.

## 2. Results

### 2.1. Identification of Rboh Gene Family Members in C. sinensis

**Figure 1:**
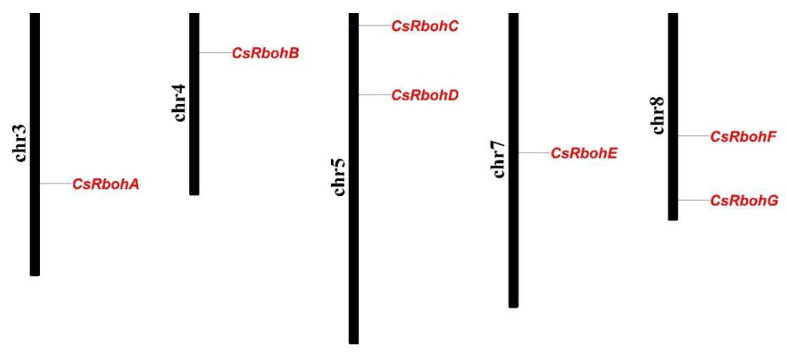
Chromosomal localization of the *CsRboh* genes. The chromosome numbers are indicated on the left of each chromosome.

A total of seven putative *CsRboh* genes were identified from the *C. sinensis* genome by HMMER and BLASTP searches. These candidate *CsRboh* genes were named as *CsRbohA*-*G* based on their localization in the *C. sinensis* genome and the widely accepted nomenclature system. *CsRboh* genes were localized on chromosomes three (*CsRbohA*), four (*CsRbohB*), five (*CsRbohC* and *CsRbohD*), seven (*CsRbohE*), and eight (*CsRbohF* and *CsRbohG*) (Figure 1). As shown in Table 1, the predicted CsRboh proteins consisted of 777–946 amino acids, with calculated molecular weights from 88.70 to 107.50 kDa, and their isoelectric points (pI) ranged from 8.57 (CsRbohD) to 9.37 (CsRbohC). For the instability index (II), only one protein (CsRbohG) was predicted as stable. The grand average of the hydropathicity (GRAVY) value of all CsRboh proteins was less than zero, suggesting that these proteins were hydrophilic [21]. In addition, the in-silico subcellular localization prediction using Plant-mPLoc indicated that all CsRboh proteins were localized in the cell membrane [22].

### 2.2. Analysis of Domain Composition, Gene Structure, and Conserved Motifs of CsRbohs

**Figure 2:**
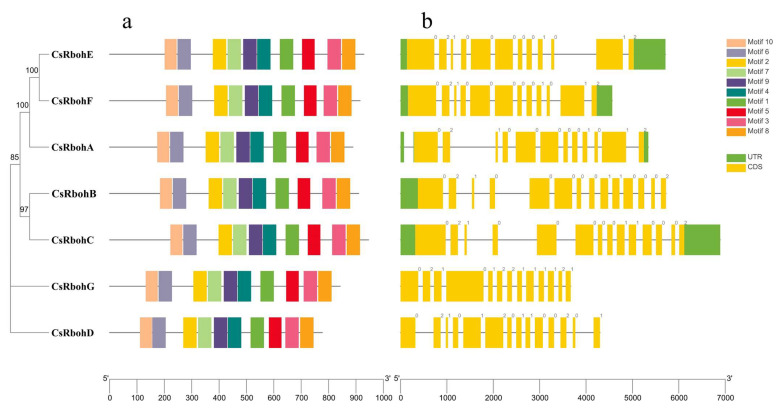
Evolutionary relationships, conserved motifs, and gene structures of *CsRboh* genes. The phylogenetic tree was constructed using MEGA-X by the Neighbour-Joining (NJ) method. (**a**) Ten types of conserved motifs in CsRboh proteins. (**b**) Gene structures of the *CsRbohs*. Exons, introns, and untranslated regions (UTRs) are indicated by yellow rectangles, gray lines, and green rectangles, respectively.

Through sequence alignment and Simple Modular Architecture Research Tool (SMART) analysis [23], we found that all the CsRboh protein sequences had four conserved domains, including NADPH_Ox and Ferric_reduct in the N-terminal region and FAD_binding_8 and NAD_binding_6 in the C-terminal region (Supplementary Figures S1 and S2). Furthermore, all the CsRboh protein sequences contained EF-hand domain and transmembrane regions except for CsRbohD. Among the ten motifs (Figure 2a), motif 10 was located in the NADPH_Ox domain region; motifs 2, 4, 7, and 9 consisted of the Ferric_reduct domain; motifs 1 and 5 were found in the FAD_binding_8 domain; and motifs 3, 5, and 8 constituted the NAD_binding_6 domain in all CsRboh proteins. Gene structure analysis revealed that the numbers of exons and introns among *CsRboh* genes varied between 12 and 14 and 11 and 13, respectively (Figure 2b). Notably, the size of *CsRboh* genes is generally determined by the size of introns. Overall, similar gene structures were found in some *CsRboh* genes clustered in the same group.

### 2.3. Phylogenetic Analysis of CsRboh Genes

**Figure 3:**
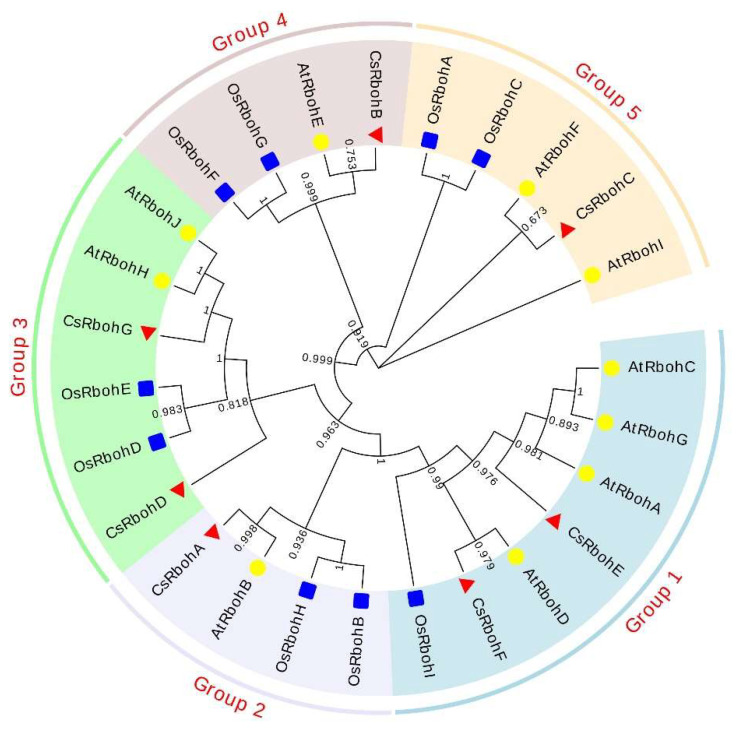
Phylogenetic tree of Rbohs in *C. sinensis*, rice, and *Arabidopsis thaliana*. The phylogenetic tree was constructed using MEGA-X by the NJ method with p-distance substitution model (gamma = 1) and 1000 bootstrap replicates.

To understand the evolutionary relationship among the Rbohs in *Citrus*, *Arabidopsis*, and rice, 26 Rboh predicted protein sequences were used to construct a phylogenetic tree (Figure 3). According to the phylogenetic tree, Rbohs were clustered into five groups, and the CsRbohs were distributed in each group. The CsRbohE and CsRbohF belonged to Group 1; CsRbohA was classified into Group 2; CsRbohD and CsRbohG were part of Group 3; CsRbohB was categorized into Group 4; CsRbohC was assigned into Group 5.

### 2.4. Prediction of Cis-Acting Elements and miRNA Target Sites

**Figure 4:**
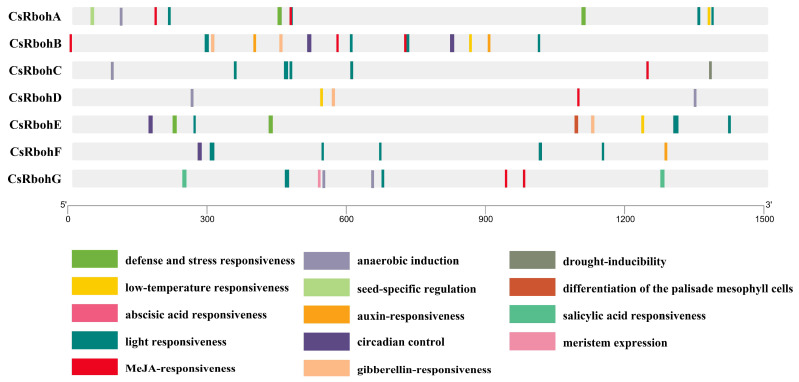
Prediction of *Cis-*acting elements in the *CsRboh* promoter regions. PlantCARE was used to identify the putative *cis*-acting element distribution in 1500 bp promoter sequences of 7 CsRbohs; *cis*-acting elements were divided into fourteen types.

*Cis-*acting elements are known to play vital roles in the regulation of gene expression [24]. To better understand *CsRboh* gene function and transcriptional regulation mechanisms, the *cis-*acting elements in 1500 bp upstream areas from the translation initiation site were predicted for all *CsRboh* genes using the PlantCARE database [25]. As presented in Figure 4, a total of fourteen representative *cis*-acting elements were selected, including five stress response elements (defense and stress responsiveness, low-temperature responsiveness, light responsiveness, anaerobic induction, and drought-inducibility), five hormone response elements (abscisic acid responsiveness, MeJA-responsiveness, auxin-responsiveness, gibberellin-responsiveness, and salicylic acid responsiveness), and four development regulation elements (seed-specific regulation, circadian control, differentiation of the palisade mesophyll cells, and meristem expression). Among these, the *cis-*acting elements of light responsiveness, MeJA-responsiveness, and abscisic acid responsiveness were highly enriched in the promoters of most *CsRboh* genes.

All the miRNAs from *Citrus* were used as query sequences to predict the target sites on *CsRboh* genes by psRNATarget with stringent settings. *CsRbohE* was targeted by csi-miR164a/b/c/d with sites in the NAD_binding_6 domain (Table 2).

### 2.5. Expression Analysis of CsRboh Genes in Different Tissues and Cold Stress Treatments

**Figure 5:**
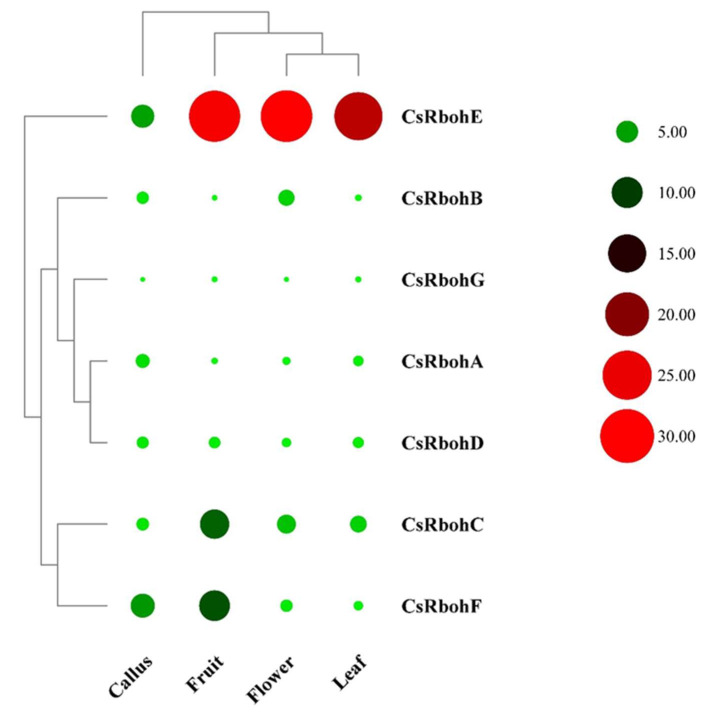
Expression patterns of the *CsRboh* genes in various tissues/organs using available RNA-seq data. The colour scale and circle size are provided with the heat map to indicate the levels of differential expression. Red represents high expression level, green represents low expression level.

**Figure 6:**
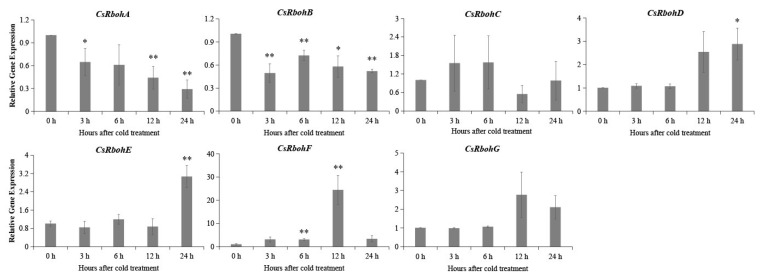
Expression patterns of the *CsRboh* genes under cold stress treatments. Asterisks indicate significant difference between control and cold treatment (* *p* < 0.05, ** *p* < 0.01).

To shed light on the possible function of *CsRboh* genes, the available RNA-seq data of the *C. sinensis* genome database (http://citrus.hzau.edu.cn/ accessed on 10 May 2021) were analyzed [26]. The results showed these *CsRboh* genes had different tissue-specific expression patterns (Figure 5). *CsRbohE* was highly expressed in all examined tissues, whereas *CsRbohG* was expressed at very low levels. In addition, the expression pattern of *CsRbohA* and *CsRbohD* genes show similar profiles in the four tissues. To explore the response of *CsRboh* genes to cold stress in *Citrus*, the expression levels of *CsRboh* genes were detected after low-temperature treatment by qRT-PCR analysis. As shown in Figure 6, the expression levels of *CsRbohA* and *CsRbohB* were significantly down-regulated within 24 h, while the transcript abundance of *CsRbohD* and *CsRbohE* was significantly increased at the 24 h time point, and *CsRbohF* was significantly upregulated at the 6/12 h time point. Furthermore, *CsRbohC* and *CsRbohG* gene expression did not differ significantly.

### 2.6. Silencing of CsRbohD in Poncirus trifoliata by VIGS

**Figure 7:**
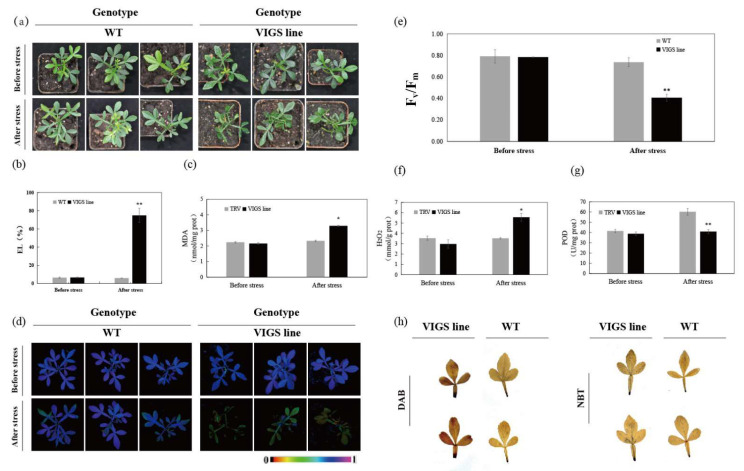
Silencing of *CsRbohD* by VIGS reduces cold tolerance in trifoliate orange. (**a**–**g**) Phenotype (**a**), EL (**b**), MDA level (**c**), Chlorophyll fluorescence imaging (**d**), F_v_/F_m_ ratio (**e**), H_2_O_2_ content (**f**), and POD activity (**g**) of control plants (TRV) and VIGS plants (TRV-CsRbohD) before and after the freezing treatment (12 h at −4 °C). (**h**) DAB and NBT staining of the leaves of trifoliate orange under cold stress. Asterisks indicate significant difference between the WT and the VIGS lines (* *p* < 0.05, ** *p <* 0.01).

Given that *CsRbohD* was significantly induced by cold stress and a low-temperature responsive element was also present in the promoter of *CsRbohD*, subsequent analyses focused on this gene by VIGS in trifoliate oranges. *CsRbohD* transcript levels were repressed in ten VIGS plants, ranging from 22% to 77%, compared to TRV empty vector control (Supplementary Figure S3). No obvious phenotypical differences were found between the TRV control plants and the VIGS lines without cold stress. However, when subjected to −4 °C treatment for 12 h, the VIGS lines exhibited more severe wilting, along with higher electrolyte leakage (EL) and malondialdehyde (MDA) contents, and weaker chlorophyll fluorescence and lower F_v_/F_m_ ratio in comparison with the TRV control plants (Figure 7a–e). Moreover, histochemical staining with nitroblue tetrazolium (NBT) and 3,3′-diaminobenzidine (DAB), together with quantitative measurement, demonstrated that ROS accumulation was much greater in the VIGS lines than in the control (Figure 7f, h). By contrast, peroxidase (POD, EC 1.11.1.7) activities in the VIGS lines were significantly lower compared with those in control (Figure 7g). These results indicate that the silencing of *CsRbohD* in trifoliate orange compromised cold tolerance.

## 3. Discussion

Rbohs are responsible for ROS generation and, thereby, are involved in regulating a diverse range of biological processes including development and stress responses in plants [3,27]. However, no comprehensive analysis of the *Rboh* gene family has been reported in *Citrus*. Here, we identified a total of seven *Rboh* genes from the *Citrus sinensis* genome (Table 1). The number of *Rboh* genes in *Citrus* is similar to previous reports of grapes [8], strawberries [12], jatropha [13], cassava [14], and pepper [16], but relatively small. Prediction programs for subcellular localization showed that the seven CsRboh proteins were localized in the cell membrane. The results are in agreement with those obtained in other studies [10,13,14,16,28], suggesting Rbohs’ potential role in the regulation of ROS generation. Notably, few Rboh proteins were predicted to localize in chloroplast thylakoid membranes or mitochondria. For instance, the FvRbohC, VvRbohB, VvRbohC2, VvRbohE, and VvRbohH proteins were found in the chloroplast thylakoid membrane [8,12], and GaRboh9, GrRboh5, GhRboh2/15, and GbRboh2/15 were found in the mitochondria [29], indicating different cellular functions. Analysis of the domain composition, gene structure, and conserved motifs of CsRbohs revealed that members of the *CsRboh* gene family were relatively conserved during evolution (Figure 2, Supplementary Figures S1 and S2).

Phylogenetic analysis demonstrated that Rbohs of *Citrus*, *Arabidopsis*, and rice were clustered into five groups, consistent with earlier reports [12,16], and the CsRbohs showed a closer evolutionary relationship to *Arabidopsis* compared with those from rice (Figure 3). In addition, most genes within the same group had similar gene structures, implying that members of the same phylogenetic subgroup may have similar functions. The available RNA-seq data showed that *CsRbohE*, clustered in the same group 1 as the *AtRbohD* gene, was highly expressed in all four tissues (Figure 5). Again, the homologous gene *AtrbohD* was expressed in all tissues of *Arabidopsis* and was involved in many physiological processes such as stomatal closure, root formation, and systemic signaling in response to diverse stresses [1,30]. Therefore, we speculate that *CsRbohE* may have a similar function to *AtRbohD*. Nevertheless, further studies are necessary to confirm this point.

Analysis with PlantCARE showed that there were various *cis-*acting elements present in the promoters of the *CsRboh* genes (Figure 4) that could be grouped into three different functional categories, namely stress response elements, hormone response elements, and development regulation elements [25]. This kind of distribution pattern of *cis*-acting elements was also reported for many other plants, such as *Arabidopsis*, rice, and cotton [10,24]. Such results indicated that *Rboh* genes function in multiple physiological processes, including development, biotic and abiotic stress responses, and hormone signaling. Moreover, it was surprising that 52.6% of *cis*-acting elements identified in the promoter regions of *CsRbohs* were related to stress response elements. Among them, the LTR motif (low-temperature responsive *cis*-acting element; CCGAAA) was found in the promoter regions of four *CsRboh* genes (*CsRbohA*, *CsRbohB, CsRbohD*, and *CsRbohE*). Thus, we investigated the responses of the *CsRboh* genes to low-temperature stress by qRT-PCR analysis (Figure 6). As expected, the four CsRboh genes were differentially expressed after low-temperature treatment. Interestingly, *CsRbohA*-*B* were up-regulated while *CsRbohD-E* were down-regulated, indicating their potential important roles in defense against cold stress as positive or negative regulators. In model plant *Arabidopsis thaliana*, the low-temperature responsive *cis*-acting element was also observed in the promoters of all *AtRboh* genes except for *AtRbohD-E*, and cold stress caused the up- and down-regulation of these *AtRboh* genes [24]. Similarly, three rice *Rboh* genes (*OsRbohD*, *OsRbohH*, and *OsRbohI*) were up-regulated after cold stress. The promoter regions of these genes contained low-temperature responsive *cis*-acting elements [24]. Collectively, the low-temperature responsive *cis*-acting elements may be involved in cold stress-induced *Rboh* gene expression.

A class of small noncoding RNAs, miRNAs, have been shown to play a key role in negatively regulating gene expression at the post transcriptional level [31]. Previous research has indicated that miR164 is involved in many biological processes including multiple stress responses and regulating shoot apical meristems, lateral root development, cell death, and fruit ripening [32,33,34,35,36]. Our results revealed that csi-miR164 may target the *CsRbohE* gene. This prediction would need to be further verified by experiments.

It is well known that Rbohs have emerged as key hubs for ROS signaling in plants [3,30]. So far, more than 56 members have been fully characterized in function [3]. However, the regulatory functions of Rbohs in response to cold stress have not been reported. Here, we silenced the *CsRbohD* gene using VIGS in trifoliate oranges, and found that silencing of *CsRbohD* greatly compromised cold tolerance (Figure 7). Considering the fact that high levels of ROS and decreased activities of antioxidant enzymes (POD) were detected in the VIGS lines, we hypothesize that a possible *CsRbohD*-ROS signal pathway induces ROS-scavenging enzyme genes under cold stress conditions.

## 4. Materials and Methods

### 4.1. Genome-Wide Identification of Citrus sinensis Rboh Family Genes

Both the *Citrus* genome and protein sequences were retrieved from databases (*C. sinensis* genome from http://citrus.hzau.edu.cn/ accessed on 10 May 2021). To identify *Citrus Rboh* genes, BLASTP was performed using ten *Arabidopsis* Rboh protein sequences retrieved from The Arabidopsis Information Resource (TAIR, https://www.arabidopsis.org/ accessed on 10 May 2021), and the Hidden Markov Model (HMM) profile of the respiratory burst NADPH oxidase domain (PF08414), ferric reductase like transmembrane component domain (PF01794), FAD-binding domain (PF08022), and ferric reductase NAD binding domain (PF08030) downloaded from the pfam (http://pfam.xfam.org/ accessed on 10 May 2021) as queries. All the putative CsRbohs were further confirmed through the Pfam database and the SMART database for conserved domains. Based to their chromosomal location, we named these genes *CsRbohA–G*. The chromosomal location map of *CsRboh* genes was displayed by the TBtool software (https://github.com/CJ-Chen/TBtools/ accessed on 10 May 2021) [37].

### 4.2. Determination of Protein Properties, Subcellular Location, Gene Structure, and Conserved Motifs

Protein properties including molecular weight, isoelectric point, instability index, and the grand average of hydropathicity were predicted using the ExPASy-ProtParam tool (http://web.expasy.org/protparam/ accessed on 12 May 2021). The subcellular localizations of CsRboh proteins were analyzed using Plant-mPLoc (http://www.csbio.sjtu.edu.cn/bioinf/plant-multi/ accessed on 12 May 2021). For the exon-intron structure determination, the coding sequences (CDS) of *CsRboh* genes were aligned with its genomic DNA sequence using TBtools software. The conserved motifs of CsRboh proteins were predicted using MEME server v 5.4.1 (http://meme-suite.org/tools/meme/ accessed on 12 May 2021) with default parameters except for the motif number set to 10 [38].

### 4.3. Multiple Sequence Alignment and Phylogenetic Analysis

Multiple alignments of the CsRboh amino acid sequences were performed using ClustalW software. The *Arabidopsis* and rice sequence data used in this study were collected from the National Center for Biotechnology Information (NCBI) (http://www.ncbi.nlm.nih.gov/ accessed on 13 May 2021). The phylogenetic tree was created using MEGA X [39] (https://www.megasoftware.net/ accessed on 10 June 2020) by Neighbor-joining algorithm with boot strap replication of 1000 times.

### 4.4. Identification of Cis-Acting Elements and Prediction of miRNA Target Sites

The 1.5 kb region upstream of the transcription start site of each *CsRboh* gene was extracted from the *C. sinensis* genome sequence and used to predict *cis*-acting elements by PlantCARE [25] (http://bioinformatics.psb.ugent.be/webtools/plantcare/html/ accessed on 20 May 2021). All known *Citrus* microRNA sequences were downloaded from miRBase database (Release 22.1) (http://www.mirbase.org/index.shtml accessed on 17 June 2021). *CsRbohs* were analyzed for the presence of miRNA target sites using psRNATarget server (http://plantgrn.noble.org/psRNATarget/ accessed on 17 June 2021) [40] with stringent parameters (to avoid false positives, maximum expectation values were set 3.5; all other parameters were used in the default setting).

### 4.5. Expression Patterns of CsRboh Genes

mRNA-seq data for different *Citrus* tissues were downloaded from the sweet orange genome annotation database (http://citrus.hzau.edu.cn/ accessed on 10 May 2021) and calculated as retrieved fragments per kilobase of transcript per million fragments mapped (FPKM) values for *CsRbohs*.

### 4.6. Plant Materials and Cold Treatments

*C. sinensis* (Valencia sweet orange) seeds were sterilized in sodium hypochlorite (10%) for 20 min and washed with distilled water three times, followed by sowing on MT medium added with 30 g L^–1^ sucrose. For cold treatments, 6-week-old seedlings were incubated at 4 °C for 0, 3, 6, 12, and 24 h, after which the leaves were harvested into liquid nitrogen and stored at −80 °C until RNA extraction. For each experiment, three biological replicates were performed, and the leaves from five seedlings were collected for each replicate.

### 4.7. RNA Extraction and Quantitative Real-Time PCR Analysis

Total RNA was extracted using Trizol reagent (TaKaRa, Dalian, China) according to the manufacturer’s instructions. The quality of the RNA was verified by gel electrophoresis and Bioanalyzer (Agilent2100). The first strand complementary DNA (cDNA) was synthesized using TransScript Reverse transcriptase (TaKaRa, Dalian, China) according to the manufacturer’s instructions. The expression of the gene encoding β-actin was used as an internal expression control. The gene-specific primers used for the qRT-PCR were listed in Supplementary Table S1. QRT-PCR was performed with CFX96 real-time PCR machine (BIO-RAD, Berkeley, CA, USA). The reaction system and procedure of qPCR were performed according to the procedures previously described [41]. The 2^−ΔΔCT^ Ct method was used to calculate the relative gene expression level across the samples [42].

### 4.8. Generation of VIGS Plants

VIGS-mediated suppression of *CsRbohD* was conducted according to previous protocols [43]. A 420 bp fragment of *CsRbohD* was amplified and inserted into the *Sma*I and *Bam*HI sites of tobacco rattle virus-based vector 2 (TRV2) to obtain the pTRV2-CsRbohD construct. The vectors pTRV1, pTRV2, and pTRV2-CsRbohD were introduced into *Agrobacterium tumefaciens* strain GV3101 by heat shock. The bacterial solutions were used to infiltrate trifoliate orange germinating seeds with emerging shoots (around 2 cm) as previously described [43]. After the infiltration, the seedlings were then rinsed with water, dried on filter paper, and transplanted to a controlled growth chamber (Percival, IA, USA) for approximately 1 month. Fully expanded leaves were collected from each plant and subjected to genomic PCR and qRT-PCR analyses, and the seedlings that exhibited silencing of *CsRbohD* were used for further analysis.

### 4.9. Cold Tolerance Assays, Physiological Measurements and Histochemical Staining

For cold tolerance assay, about 1-month-old VIGS plants and WT plants were exposed to −4 °C for 12 h. Growth performance and chlorophyll fluorescence imaging of the plants were analyzed, while leaves were harvested for physiological analyses or histochemical staining. The MDA and H_2_O_2_ levels and POD activities were determined using corresponding assay kits (Nanjing Jiancheng Bioengineering Institute, China). MDA assay was performed using the thiobarbituric acid method and calculated by the absorbance of TBA reactive substances at 532 nm. H_2_O_2_ assay was based on the oxidative polymerization of molybdic acid into a complex compound which can be determined at 405 nm. The MDA or H_2_O_2_ contents were expressed as nmol mg^−1^ protein or mmol g^−1^ protein, respectively. One unit of POD activity was defined as the amount of enzyme required to the formation of 1 μmol product per minute. EL was calculated based on previous reports [44], and F_v_/F_m_ ratios were measured using Imaging WinGegE software. Furthermore, histochemical staining was used for the in vivo detection of H_2_O_2_ and O_2_^−^ with DAB and NBT, respectively [45].

### 4.10. Statistical Analysis

Cold treatment was repeated at least three times, with three replicates for each line and time point. All data, expressed as mean ± SD, were analyzed by SPSS 25.0 software (IBM Corp., Armonk, NY, USA). Analysis of variance (ANOVA) was used to compare the statistical difference based on Fisher’s least significant difference test at the significance levels of *p* < 0.05 (*), and *p* < 0.01 (**).

## 5. Conclusions

In our study, seven *Rboh* genes (*CsRbohA*~*CsRbohG*) were identified in the genome of *C. sinensis* and clustered into five groups. Gene structure, motif distribution pattern, and *cis*-acting element analysis indicated the conservation and divergence of *CsRboh* genes. Csi-miR164 was predicted to target the *CsRbohE* gene. Five *CsRboh* genes were responsive to low-temperature treatment. Functional and physiological analyses suggested that the *CsRbohD* gene is involved in response to cold stress. As a whole, our results lay a foundation for further functional characterization of *CsRbohs*, and provide key candidate proteins for genetic improvement of *Citrus*.

## Figures and Tables

**Table 1:** The details of the seven predicted *CsRboh* genes identified in the current study.

Gene Name	Locus Number	Amino Acids Length (aa)	Molecular Weight (kDa)	Isoelectric Point	Instability Index	GRAVY	Subcellular Localization Predicted
*CsRbohA*	Cs3g14240	889	101.05	9.14	41.05	−0.266	Cell membrane
*CsRbohB*	Cs4g06920	910	103.35	9.19	48.25	−0.274	Cell membrane
*CsRbohC*	Cs5g02940	946	107.50	9.37	50.27	−0.263	Cell membrane
*CsRbohD*	Cs5g11890	777	88.70	8.57	49.93	−0.082	Cell membrane
*CsRbohE*	Cs7g19320	929	105.02	8.68	43.94	−0.352	Cell membrane
*CsRbohF*	Cs8g12000	915	103.53	9.11	42.82	−0.305	Cell membrane
*CsRbohG*	Cs8g17640	842	96.61	9.11	39.68	−0.170	Cell membrane

**Table 2:** List of *CsRboh* genes with putative miRNA target sites.

Gene Name	Locus Number	Predicted miRNA Target Sites	miRNA Length	Expectation	UPE
*CsRbohE*	Cs7g19320	csi-miR164a	21	3.5	23.655
*CsRbohE*	Cs7g19320	csi-miR164b	21	3.5	23.655
*CsRbohE*	Cs7g19320	csi-miR164c	21	3.5	23.655
*CsRbohE*	Cs7g19320	csi-miR164d	21	3.5	23.655

## Data Availability

Not applicable.

## Supplementary Materials

The following figures and a table are available online at https://www.mdpi.com/article/10.3390/ijms23020648/s1, Figure S1: Alignment of the CsRboh protein sequences, Figure S2: Domain compositions of CsRboh proteins by SMART analysis, Figure S3: Molecular characterization of the VIGS plants by genomic PCR and qRT-PCR, Table S1: Primers used in this study.
