# Supplementary material for: Genome Wide Identification of Respiratory Burst Oxidase Homolog (Rboh) Genes in Citrus sinensis and Functional Analysis of CsRbohD in Cold Tolerance
Source: Int J Mol Sci. 2022 Jan 7;23(2):648. doi: 10.3390/ijms23020648 (PMC8776138; doi:10.3390/ijms23020648)
Supplement: Supplementary file 1 [file ijms-23-00648-s001.zip › Supplementary Figure S1-3.pdf]

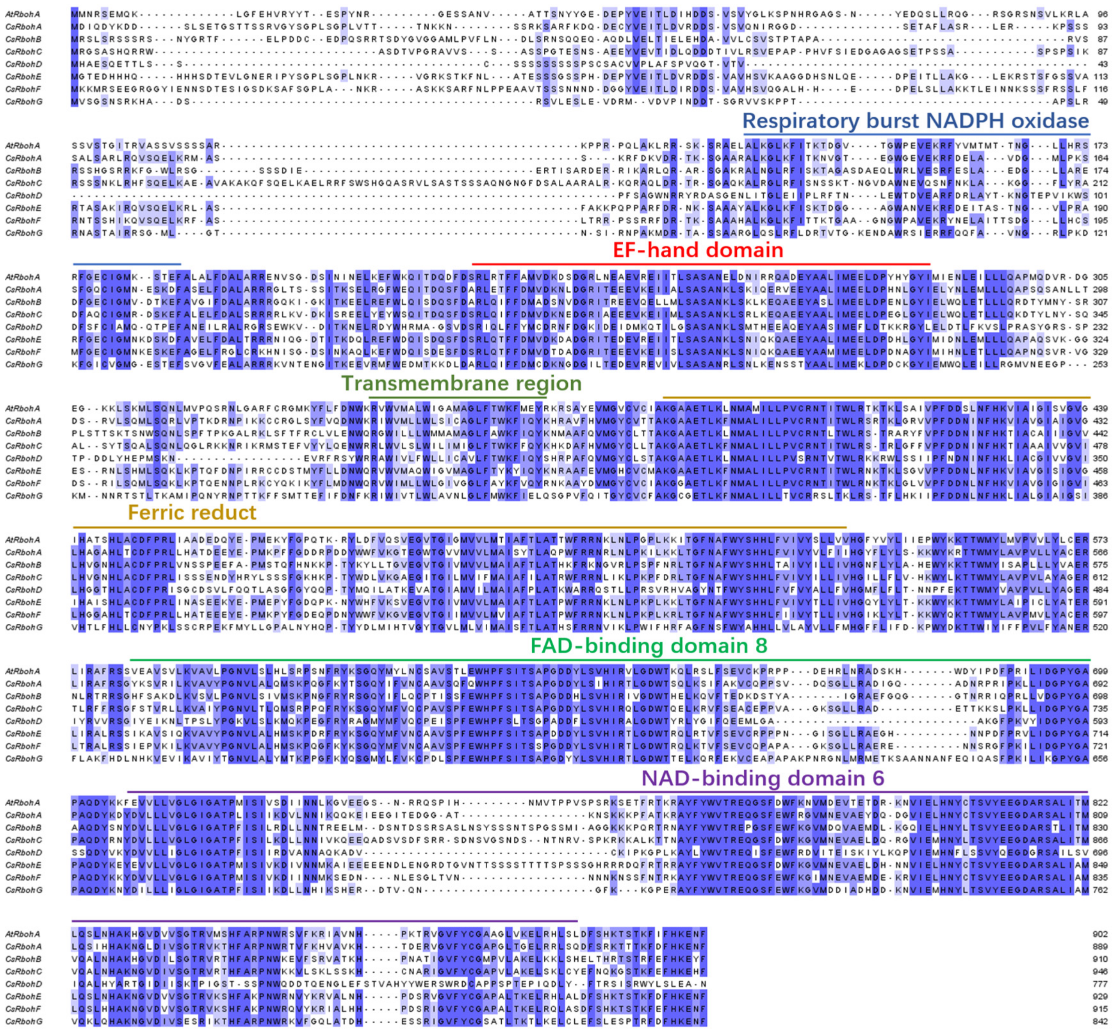

Supplementary Figure S1. Alignment of the CsRboh protein sequences. The *Arabidopsis* AtRbohA is also included in this analysis. Conserved domains in alignment are highlighted with colored lines, and their names are given above the conserved regions.

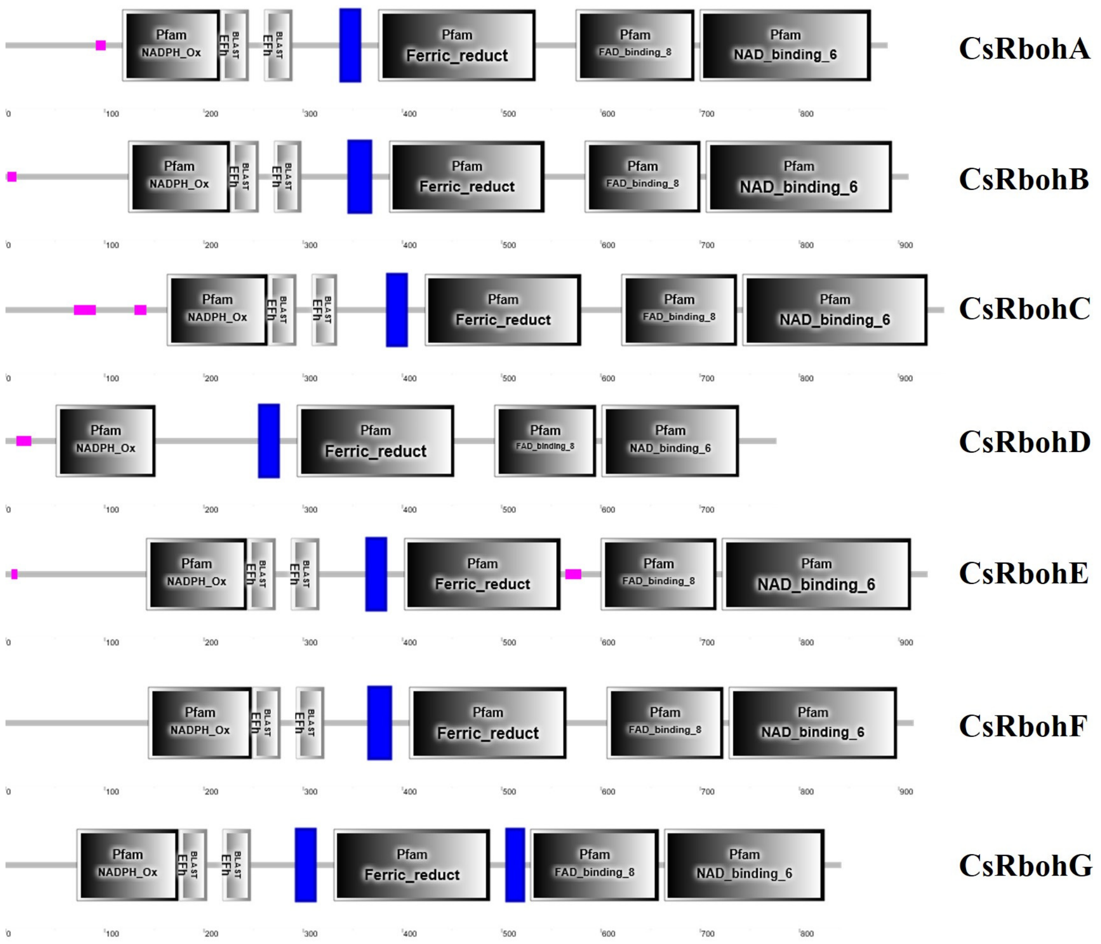

Supplementary Figure S2. Domain compositions of CsRboh proteins by SMART analysis. Only major domains (NADPH_Ox, Ferric_reduct, FAD_binding_8, and NAD_binding_6) were presented here. Blue box in figure indicates the trans-membrane domain while pink indicates low complexity region.

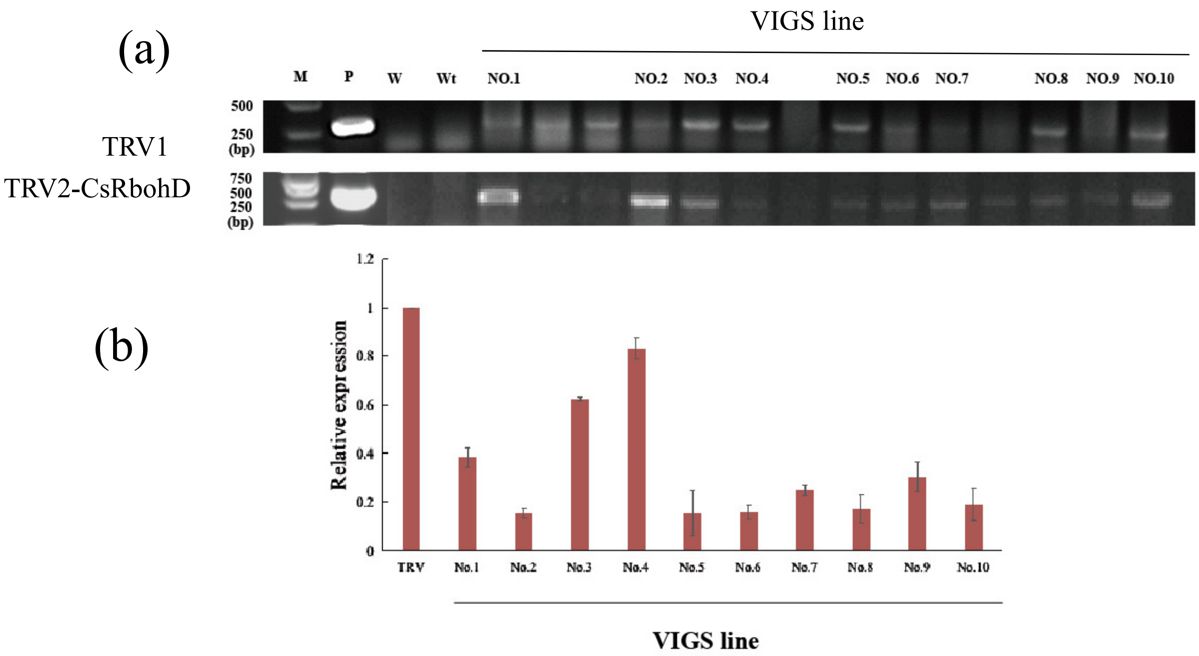

Supplementary Figure S3. Molecular characterization of the VIGS plants by genomic PCR and qRT-PCR. (a) Characterization of positive VIGS plants by genomic PCR amplification. (b) Analysis of *CsRbohD* expression in the control (TRV) and VIGS plants using qRT-PCR.
